# Endogenous and Exogenous Vanilloids Evoke Disparate TRPV1 Activation to Produce Distinct Neuronal Responses

**DOI:** 10.3389/fphar.2020.00903

**Published:** 2020-06-12

**Authors:** Rakesh Kumar, Matan Geron, Adina Hazan, Avi Priel

**Affiliations:** Institute for Drug Research (IDR), School of Pharmacy, The Faculty of Medicine, The Hebrew University of Jerusalem, Jerusalem, Israel

**Keywords:** TRPV1, endovanilloids, exovanilloids, capsaicin, bradykinin, Gq- GPCR, nociceptors, inflammatory pain

## Abstract

Neuronal signals are processed along the nociceptive pathway to convey discriminative information, which would manifest in the produced pain sensation. The transient receptor potential vanilloid 1 (TRPV1), an important signaling complex in nociceptors termini, is activated by different noxious stimuli that underlie distinct pain sensations. For example, while endovanilloids are associated with inflammatory pain and hypersensitivity through TRPV1 activation, the exovanilloid toxin, capsaicin, evokes an acute pain by activating this channel. Differences in the TRPV1 activation profile evoked by exogenous and endogenous vanilloids were suggested to underlie this disparity in pain sensations. However, the cellular processes that lead to these differences in pain sensation mediated by the same channel are not fully understood. Here, we sought to describe the neuronal response of TRPV1-expressing nociceptors to exo-and endovanilloids. To this end, we performed current-clamp recordings in rat trigeminal neurons exposed to either capsaicin or intracellular endovanilloids produced downstream of the bradykinin receptor BK2. Our results show that lipoxygenase metabolites generate persistent TRPV1-dependent action potential firing while capsaicin evokes robust depolarization and high-frequency firing that is quickly terminated by depolarization block. Additionally, we found that a weak TRPV1 activation prolongs action potential firing. Overall, our results indicate different firing patterns evoked by inflammatory mediators and capsaicin *via* TRPV1 that correlate with the respective subsequent pain sensation. These findings also suggest that differences in neuronal activation stem from the variable degree of TRPV1 activation they produce.

## Introduction

TRPV1 has been identified as an essential component of the nociceptive mechanisms underlying different pain conditions and pain sensations (Davis et al., 2000; Patapoutian et al., 2009; Mishra et al., 2011; Fattori et al., 2016). This non-selective cation channel, which is mainly found in nociceptors is a polymodal receptor that detects a wide array of noxious stimuli such as heat (>42°C), protons (low pH), plant, and animal toxins, as well as bioactive lipids (Julius, 2013). TRPV1 activators engage diverse binding domains producing a modulatory effect on this multi-steric channel (Winter et al., 2013). One main binding domain is the intracellular vanilloid binding site (VBS), located between S3 and S4, with which both plant toxins and bioactive lipids interact (Jordt and Julius, 2002). Vanilloid plant toxins, such as the prototypical capsaicin (found in chili peppers), activating TRPV1, and produce acute pain (Yang et al., 2015). In contrast, TRPV1 bioactive lipids are endogenous vanilloids mainly produced during inflammation (Levine and Alessandri-Haber, 2007; Julius, 2013). This TRPV1 activation following injury was shown to be essential for heat hyperalgesia and pain hypersensitivity (Caterina et al., 2000; Shin et al., 2002). Thus, TRPV1 mediates both the acute burning pain following the ingestion of hot peppers and the dull and prolonged pain as well as hypersensitivity that often accompany inflammation.

The endovanilloid lipids, such as 12-Hydroeicosatetraenoic acid (12-HETE), are products of arachidonic acid (AA) metabolism by lipoxygenases (LOX), downstream of the Gq/GPCR pathway (Hwang et al., 2000; Starowicz et al., 2007; Veldhuis et al., 2015; Kumar et al., 2017). Thus, different components of the inflammatory soup such as bradykinin and histamine indirectly activate TRPV1 by inducing the Gq signaling cascade through their respective receptors co-expressed with TRPV1 in nociceptors (Shin et al., 2002; Shim et al., 2007). PKC is also activated in this signaling pathway and is essential for endovanilloid activity as it phosphorylates and sensitizes TRPV1 (Premkumar and Ahern, 2000; Kumar et al., 2017). Also, LOX metabolites are highly labile as they are quickly reduced to products with reduced potency (Hwang et al., 2000; Starowicz et al., 2007). Thus, endovanilloids are components of a highly regulated machinery essential for the initial recognition and subsequent healing of tissue injury, in contrast to exovanilloid toxins that provide the evolutionary advantage of evoking a deterring intense pain.

We previously showed that endovanilloids produced through Gq signaling evoke reduced open probability in TRPV1 and thus a weak channel activation in comparison to capsaicin (Kumar et al., 2017). The different properties of TRPV1 activation by exogenous and endogenous vanilloids were suggested to underlie the distinct pain sensations experienced during acute and inflammatory settings (Kumar et al., 2017). Indeed, it was suggested that while endovanilloids evoke prolonged action potential firing in nociceptors, exovanilloids produce a short burst of action potentials followed by a depolarization block, a cessation of firing despite suprathreshold membrane potential due to the inactivation of voltage-gated sodium (Na_V_) channels (Liu et al., 2001; Shin et al., 2002; Blair and Bean, 2003). However, these observations were obtained using different experimental settings for endo- and exovanilloids. Moreover, in both cases, recordings were performed in ruptured cells where intracellular signaling (i.e., Gq signaling) is disrupted. Thus, exactly how these two types of vanilloids exert a different effect in neurons remains to be elucidated.

Here, we sought to further describe the neuronal response of TRPV1 expressing nociceptors to exo- and endovanilloids. To this end, we performed electrophysiological recordings in perforated trigeminal neurons in which the intracellular signaling apparatus is preserved. We show that endovanilloids produced downstream to bradykinin receptor activation evoke prolonged action potentials firing, which is TRPV1 dependent, while capsaicin-evoked firing is short-lived. Firing evoked by bradykinin requires the generation of endovanilloids catalyzed by PLC and LOX in the Gq signaling pathway. Also, we found that the lower depolarization induced by endovanilliods could underlie the prolonged firing as lower capsaicin concentrations partially emulate this firing pattern. Overall, our results indicate that the highly regulated, reduced activation of TRPV1 by endovanilloids produced by LOX enables prolonged firing in nociceptors in contrast to the robust firing quickly followed by depolarization block obtained following the application of the highly efficacious capsaicin. Thus, these findings demonstrate that different neuronal activation patterns could be evoked through the same binding site in TRPV1, potentially encoding different pain sensations.

## Materials and Methods

### Neuronal Cell Culture and Viral Infection

The Institutional Animal Care Committee of The Hebrew University approved all procedures that involved the use of animals. Acute culture of rat TG neurons (n = 42) was performed as previously described (Bohlen et al., 2010). In brief, dissected ganglia from two to four rat pups (P0–3) were collected in ice-cold DPBS (with Ca^2+^ and Mg^2+^) (Sigma-Aldrich, St. Louis, MO, USA), followed by digestion with 0.025% collagenase type IV (Worthington Biochemical Corp., Lakewood, NJ, USA) and then 0.25% Trypsin (Sigma-Aldrich, St. Louis, MO, USA). After enzymatic digestion, ganglia were triturated with fire-polished Pasteur glass pipettes of decreasing tip diameter. Cells were re-suspended in Dulbecco's modified Eagle's medium (DMEM, Gibco, Waltham, MA, USA) supplemented with 10% fetal bovine serum (FBS, Gibco), 1% penicillin-streptomycin (Gibco), and 25 mM 4-(2-hydroxyethyl)-1-piperazineethanesulfonic acid (HEPES; pH 7.3; Gibco, Waltham, MA, USA) (hereafter: complete DMEM) and plated on Poly-d-Lysine (PDL; Sigma, St. Louis, MO, USA) coated glass coverslips. Cells were allowed to recover for 3 h at 37°C in a 5% CO_2_ atmosphere before patch-clamp experiments. Dissociated rat TG neurons (n = 4) were infected with 0.5 ng/ml adeno-associated virus vector, serotype 2 (rAAV2) containing hSyn-HA-hM3D(Gq)-IRES-mCitrine or hSyn-EGFP (University of North Carolina Vector Core, Chapel Hill, NC, USA), in a complete media supplemented with 50 ng/ml nerve growth factor (NGF) (Alomone Labs, Jerusalem, Israel) and 1 ng/ml glial cell-derived neurotrophic factor (GDNF) (Alomone Labs, Jerusalem, Israel) for one week before electrophysiological analysis. Infected neurons were selected based on their fluorophore expression.

### Electrophysiology

#### Current-Clamp Recordings

Membrane voltages were recorded under the current-clamp mode (I = 0) using an Axopatch 200B patch-clamp amplifier (Molecular Devices, Sunnyvale, CA, USA). Membrane voltages were digitized using a Digidata 1440A interface board and pCLAMP 10.6 software (Molecular Devices, Sunnyvale, CA, USA) at room temperature. Sampling frequency was set to 20 kHz and low-pass filtered at 5 kHz. Patch electrodes were fabricated from borosilicate glass using the P1000 Micropipette Puller (Sutter Instrument) and fire-polished using the Microforge MF-900 (Narishige, East Meadow, NY, USA) to a resistance of 2 to 4 MΩ for whole-cell and perforated patch recordings. Perforated patch or standard whole-cell configurations of the patch-clamp technique were obtained in voltage-clamp mode before proceeding to current-clamp recording mode. The perforated patch configuration was carried out as previously described (Priel and Silberberg, 2004). Briefly, the pipette solution constituted (mM): 75 K_2_SO_4_, 55 KCl, 5 MgSO_4_, 10 HEPES, adjusted to pH 7.2 with KOH. Nystatin (Sigma, St. Louis, MO, USA) was used for patch perforation at a working concentration of 180 µM. To this end, it was dissolved in Dimethyl Sulfoxide (DMSO; Sigma, St. Louis, MO, USA) to obtain a 55 mM stock solution, which following 1 min ultra-sonication was diluted in pipette solution to obtain a working solution of 180 µM. Nystatin solutions were freshly prepared in the dark every 2 h. Only cells with a series resistance of ≤15 MΩ were used for analysis. The extracellular solution contained (mM): 140 NaCl, 2.8 KCl, 2 MgSO_4_, 1.8 CaCl_2_, 10 HEPES, 10 D-glucose, adjusted to pH 7.4 with NaOH (Ringer solution). Once the perforated-patch or the standard whole-cell configuration was established, cells were continuously superfused with extracellular solutions *via* the ValveBank perfusion system (AutoMate Scientific, Berkeley, CA, USA). Cells were incubated with U73122 for 5 minutes before recording, whereas other drugs and chemicals were applied as indicated in the figures. For the standard whole-cell configuration pipette solution contained (mM): 130 potassium-gluconate (KC_6_H_11_O_7_), 2 MgSO_4_, 6 KCl, 10 NaCl, 10 HEPES, 5 MgATP, 0.5 Li_2_GTP, adjusted to pH 7.4 with KOH. Small TG neurons, (~25 μm), shown to be nociceptors based on their capsaicin-sensitivity (Cardenas et al., 1995; Le Pichon and Chesler, 2014) were selected for recording. Small TG neurons with stable (<10% variation) resting membrane potentials (≤−50 mV), action potentials with overshoots >40 mV, and amplitudes >85 mV were analyzed (Blair and Bean, 2003; Cummins et al., 2009; Gudes et al., 2015). Only neurons with a threshold of −35 ± 3 mV, determined according to the first action potential elicited under the fast current-clamp mode of a single ramp of depolarizing current injection (500 ms; 0–150 pA) (Gudes et al., 2015), were selected for analysis. In perforated current-clamp recordings, junction potential was calculated to be 9.3 mV (using Clampfit 10.6). The membrane potential was corrected accordingly. The phase plots of dV/dt vs. membrane voltage were formed by plotting the rate of change of membrane potential to time (dV/dt), following the application of bradykinin or capsaicin, as a function of membrane potential. The first two evoked APs in each cell were analyzed. Maximum upstroke velocity was obtained from (dV/dt) Max.

#### Voltage-Clamp Recordings

Recordings from TG neurons were performed as previously described (Kumar et al., 2016). Briefly, membrane currents were recorded under the voltage-clamp using an Axopatch 200B patch-clamp amplifier (Molecular Devices, Sunnyvale, CA, USA). Membrane currents were digitized using a Digidata 1440A interface board and pCLAMP 10.6 software (Molecular Devices, Sunnyvale, CA, USA) with sampling frequency set to 10 kHz and were low-pass filtered at 5 kHz. The holding voltage was −60 mV. All experiments were carried out at room temperature.

### Data Analysis

The electrophysiological analysis was performed using pCLAMP 10.6 software (Molecular Devices, Sunnyvale, CA, USA). All statistical data were calculated using SigmaPlot 11 (Systat Software, San Jose, CA, USA) and Prism 7 (Graphpad Software, La Jolla, CA, USA) software. Student's *t*-test and ANOVA were used to determine statistical significance.

### Drugs and Chemicals

All salts and buffers were purchased from Sigma-Aldrich (St. Louis, MO, USA). Capsaicin (Cap), Capsazepine (CPZ), U73122, and Bisindolylmaleimide I (BIM I; GF 109203X) were purchased from Tocris Bioscience (Bristol, UK). Bradykinin (Bk), Histamine dihydrochloride (His), Nordihydroguaiaretic acid (NDGA), Bupivacaine hydrochloride (bupivacaine), and Clozapine N-oxide (CNO) were purchased from Sigma-Aldrich (St. Louis, MO, USA). All drugs were dissolved according to manufacturer protocol.

## Results

### Inflammatory Mediators and Exovanilloids Evoke Distinct Firing Patterns

**Figure 1:**
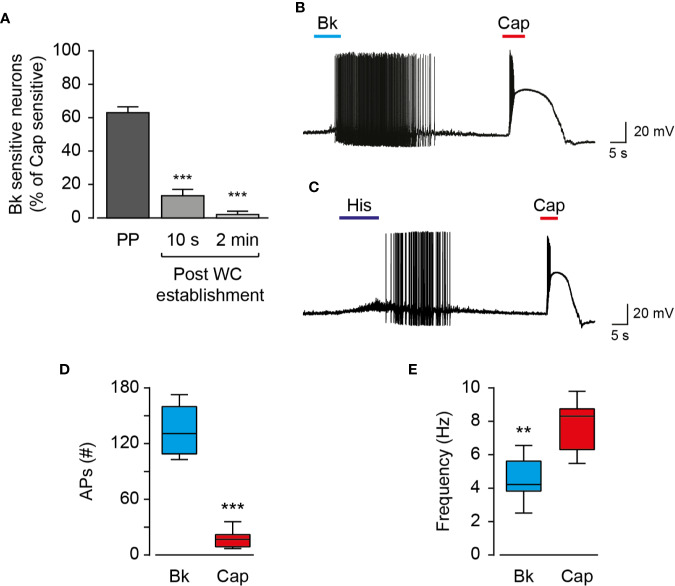
Endo- and exovanilloids evoke distinct patterns of neuronal activation. **(A)** Bar diagram showing the average (± SEM) percentage of bradykinin (“Bk”; 0.5 µM) sensitive nociceptors (n = 6–27) at different configurations of the patch-clamp technique, normalized to the percentage of capsaicin (“Cap”; 0.2 µM) -sensitive cells. Sensitive cells were defined by the detection of agonist-evoked action potentials. Statistical significance between perforated whole-cell (“Perforated”; n = 27) and the whole-cell (“WC”; n = 6) configurations were determined using ANOVA with multiple comparisons, where *** represents p ≤ 0.001. **(B)** Representative current-clamp recording (I = 0) from rat TG neurons using the perforated whole-cell configuration in response to capsaicin (“Cap”; red bar) and bradykinin (“Bk”; cyan bar) (n = 12). Bradykinin (0.5 µM) was applied for 15 s followed by capsaicin (0.2 µM) application for 5 s. **(C)** Representative current-clamp recording (I = 0) from rat TG neurons using the perforated whole-cell configuration in response to capsaicin (“Cap”; red bar) and histamine (“His”; dark blue bar) (n = 4). Histamine (20 µM) was applied for 15 s followed by capsaicin (0.2 µM) application for 5 s. **(D)** Box and whiskers plot shows the number of evoked action potentials (APs (#)) in response to bradykinin (0.5 µM) or capsaicin (0.2 µM) (n = 12). ***p ≤ 0.001, paired Student *t*-test. **(E)** Box and whiskers plot shows the firing rate (Frequency) in response to bradykinin (0.5 µM) or capsaicin (0.2 µM) (n = 12). **p ≤ 0.01 paired Student's *t*-test.

**Figure 2:**
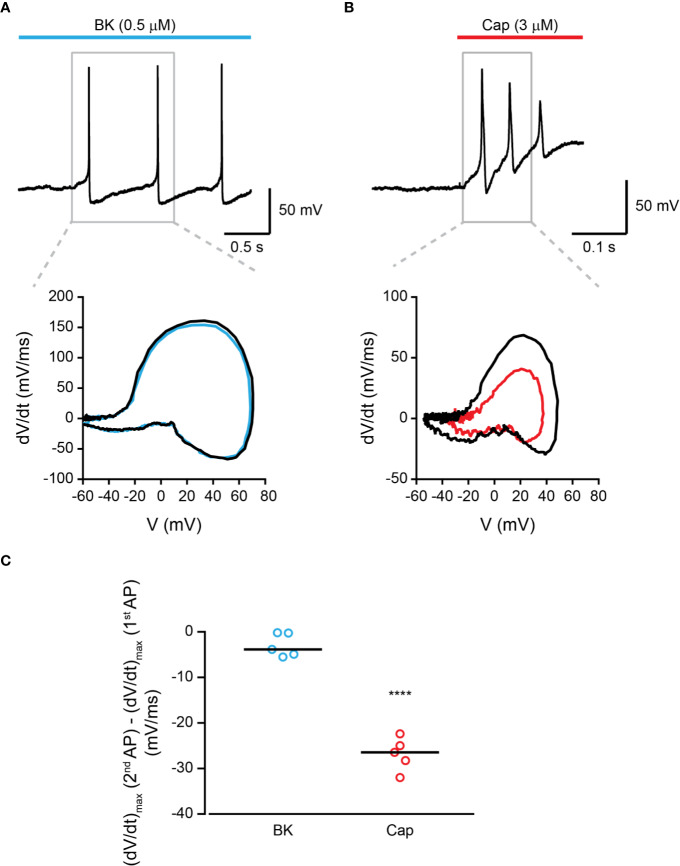
Bradykinin-evoked activation of TRPV1 produces repetitive AP firing. **(A)** *Top:* Current-clamp recording (I = 0) from small-diameter dissociated rat trigeminal ganglion (TG) TRPV1+ neurons (P0) using the perforated whole-cell configuration of the patch-clamp technique. Representative trace of the first three APs following the application of bradykinin (“Bk”; cyan bar; 0.5 µM) is shown. *Bottom:* Phase plots of the rate of change of the membrane potential (dV/dt) during the first (black trace) and second (cyan trace) APs vs. membrane potential (V). **(B)** *Top:* Current-clamp recording (I = 0) from small-diameter dissociated rat trigeminal ganglion (TG) TRPV1+ neurons (P0) using the perforated whole-cell configuration of the patch-clamp technique. Representative trace of the first three APs following the application of capsaicin (“Cap”; red bar; 3 µM) is shown. *Bottom:* Phase plots of the rate of change of the membrane potential (dV/dt) during the first (black trace) and second (red trace) APs vs. membrane potential (V). **(C)** Mean/scatter-dot plot representing the difference in the maximal velocity (dV/dt) of the upstroke of the AP between the second and first APs evoked by either bradykinin (“Bk”; 0.5 µM; cyan circles) or capsaicin (“Cap”; red circles; 3 µM) (n = 5). ****p ≤ 0.0001, unpaired Student's *t*-test.

Endo- and exovanilloids produce different activation profiles of TRPV1 expressed in a heterologous expression system (Kumar et al., 2017). To characterize the neuronal response to endogenous and exogenous vanilloids (i.e., capsaicin), we performed recordings in acutely dissociated trigeminal neurons from neonatal rats. In contrast to exovanilloids, endovanilloids are highly lipophilic, and thus their ability to cross the plasma membrane and reach the intracellular VBS in the TRPV1 channel when applied extracellularly is reduced as they can be trapped in the lipid bilayer (Hwang et al., 2000; Starowicz et al., 2007; Ursu et al., 2010). Under physiological conditions, endovanilloids were suggested to be LOX metabolites, produced downstream of Gq/GPCR (Hwang et al., 2000; Veldhuis et al., 2015). This pathway can be activated through receptors such as the bradykinin receptor BK2 and the histamine receptor H1 that were shown to co-express with TRPV1 (Carr et al., 2002; Shin et al., 2002; Pethő and Reeh, 2012). Thus, throughout this study, we sought to induce cell-autonomous endovanilloid production by activating the Gq/GPCR pathway using the inflammatory mediators bradykinin and histamine. Accordingly, we employed the perforated whole-cell configuration of the patch-clamp technique, which in contrast to the standard whole-cell configuration, preserves the cellular milieu and allows signaling events downstream of GPCR activation (Knauer and Yoshida, 2019). Indeed, in perforated patch recordings, 18 out of 27 TRPV1-expressing nociceptors responded to bradykinin, whereas only 1 out of 6 were bradykinin- sensitive in whole-cell configuration recordings (**Figure 1A**). Of note, six out of the 33 small diameter neurons recorded under the perforated configuration were sensitive to bradykinin but not to capsaicin. Also, just four of 36 capsaicin-responsive nociceptors responded to histamine in the perforated configuration. This low rate of co-expression is expected as H1R expression in TRPV1^+^ neurons is scarce, whereas TRPV1 is expressed in most H1R^+^ neurons (Roberson et al., 2013). As shown in **Figures 1B, C**, while exposure to bradykinin or histamine evoked a long train of action potentials, exposure to capsaicin (0.2 µM) resulted in a robust depolarization with few action potentials that were followed by an immediate depolarization block. While the persistent neuronal activation by bradykinin allowed a higher number of action potentials firing, capsaicin evoked a higher firing frequency (**Figures 1D, E**). Therefore, our analysis indicates that inflammatory mediators and capsaicin (0.2 µM) produce distinctive neuronal firing patterns as both bradykinin and histamine evoke a more prolonged neuronal activation, albeit with reduced firing rate in TRPV1-positive nociceptors as compared to capsaicin. In order to further analyze the TRPV1-induced firing patterns, we compared the rate of change of membrane voltage as a function of membrane voltage, dV/dt vs. V, between the first and second APs. As shown in **Figure 2**, we found that sequential capsaicin-evoked APs present a significant decrease in maximal upstroke velocity [(dV/dt)max]. As (dV/dt)max is determined by the number of available Na_V_ channels (i.e., non-inactivated), this indicates that the fast and robust depolarization evoked by capsaicin causes the gradual inactivation of these channels until depolarization block is reached (Gudes et al., 2015). In contrast, bradykinin-evoked APs are mostly unchanged, suggesting the full recovery of Na_V_ channels between APs.

### Bradykinin-Evoked Firing Is Mediated by TRPV1 and Requires LOX Metabolites

**Figure 3:**
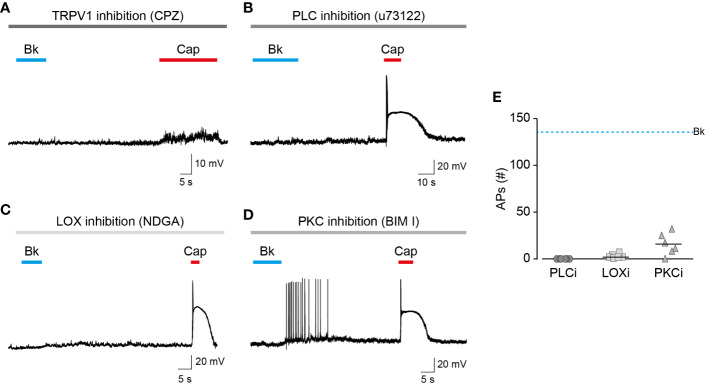
Endovanilloids produced by LOX evoke TRPV1-dependent nociceptor firing. Current-clamp recordings (I = 0) from small-diameter dissociated rat trigeminal ganglion (TG) neurons (P0) using the perforated whole-cell configuration of the patch-clamp technique. **(A)** Representative recording in which bradykinin (0.5 µM) was applied for 15 s followed by capsaicin (1 µM) application for 20 s, in the continuous presence of capsazepine (“CPZ”; 40 µM; grey bar) (n = 6). **(B)** Representative recording in which bradykinin (0.5 µM) was applied for 15 s followed by capsaicin (0.2 µM) application for 5 s, in the continuous presence of U73122 (3 µM; bar) (n = 6). **(C)** Representative recording in which bradykinin (0.5 µM) was applied for 10 s followed by capsaicin (0.2 µM) application for 5 s, in the continuous presence of nordihydroguaiaretic acid (“NDGA”; 10 µM; bar) (n = 7). **(D)** Representative recording in which bradykinin (0.5 µM) was applied for 10 s followed by capsaicin (0.2 µM) application for 5 s, in the continuous presence of bisindolylmaleimide I (“BIM I”; 10 µM; grey bar) (n = 6). **(E)** Mean/scatter-dot plot representing the number of action potentials (APs) evoked by bradykinin while PLC (“PLCi”; dark grey circles), LOX (“LOXi”; light grey squares) or PKC (“PKCi”; grey triangles) are inhibited (n = 6–7). The dashed line indicates the average number of action potentials evoked by bradykinin in the absence of inhibitors.

The bradykinin receptor, BK2, was previously shown to cause membrane depolarization by activating ion channels other than TRPV1 (Katanosaka et al., 2008). This includes the activation of ANO1, sensitization of TRPA1, and inhibition of Kv7 channels (Liu et al., 2010). However, as was previously shown by others (Shin et al., 2002), the application of the competitive TRPV1 antagonist, capsazepine, completely abolished action potential firing evoked by bradykinin as well as capsaicin (**Figure 3A**). Like other GPCRs, BK2 can activate various G protein signaling pathways (i.e., Gi, and Gq) (Miyamoto et al., 1997; Sandhu et al., 2019). In contrast, the engineered Gq-DREADD (hM3D(Gq)) receptor exclusively interacts with Gq proteins when it is bound to its selective activator, clozapine-N-oxide (CNO) (Roth, 2016). Nonetheless, in dissociated TRPV1-positive TG neurons infected with adeno-associated virus-carrying Gq-DREADD (hM3D(Gq)), CNO evoked a comparable response to that evoked by bradykinin in EGFP-infected neurons (**Figure S1**). Thus, activation of the promiscuous BK2 mimics that of the Gq-DREADD that specifically induces Gq signaling, suggesting that other pathways have a minor effect on BK-evoked nociceptive firing. Inhibiting PLC, which initiates the signaling cascade downstream Gq, by incubating neurons with U73122, produced an attenuated response to bradykinin while the capsaicin response remained intact (**Figure 3B**). This further demonstrates that the neuronal response to bradykinin is Gq-dependent. To confirm the role of endovanilloids, produced by LOX in the neuronal response to bradykinin, neurons were incubated with the broad LOX inhibitor, nordihydroguaiaretic acid (NDGA). Similarly to PLC inhibition, neurons in which LOX was inhibited presented a diminished response to bradykinin, whereas the response to capsaicin was unaffected (**Figure 3C**). Thus, neuronal activation by bradykinin is mediated by endovanilloids, which are LOX metabolites. Sensitization of TRPV1 by PKC phosphorylation was shown to be essential to the activation of the channel by endovanilloids (Bhave et al., 2003; Kumar et al., 2017). Indeed, as shown in **Figure 3D**, inhibition of PKC by bisindoylmaleimide I (BIM) resulted in a reduced action potential firing evoked by bradykinin, thus demonstrating that PKC-mediated sensitization potentiates the neuronal response to endovanilloids. Together, these results confirm that the bradykinin-evoked prolonged firing in these neurons occurs through the activation of TRPV1 by endovanilloids produced downstream of the Gq signaling pathway (**Figure 3E**).

### Reduced TRPV1 Current and Membrane Depolarization Result in Prolonged AP Firing

**Figure 4:**
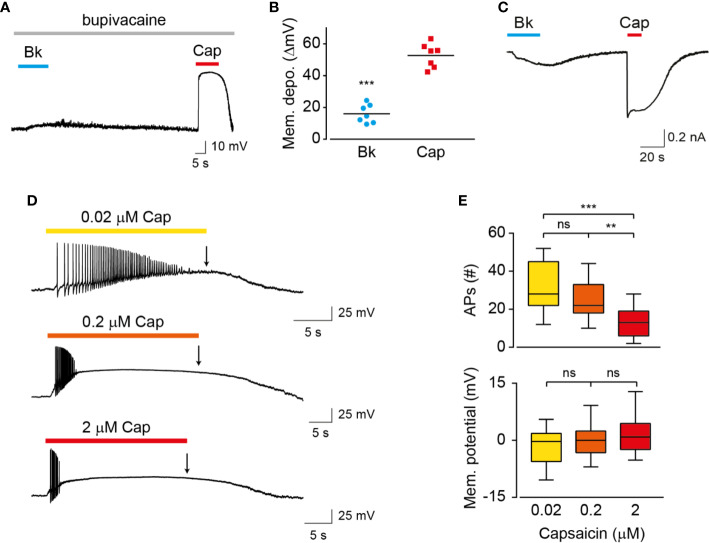
Endo- and exovanilloids evoke distinct neuronal membrane depolarization. **(A)** Representative current-clamp recording (I = 0) from rat TG neurons using the perforated whole-cell configuration in response to bradykinin (“Bk”; 1 µM; cyan bar and circles) that was applied for 15 s followed by capsaicin (“Cap”; 3 µM; red bar and squares) application for 15 s in the continuous presence of bupivacaine (300 µM; light grey bar) (n = 7). **(B)** Mean/scatter-dot plot representing the evoked membrane depolarization (“Mem. depo.”; ΔmV) in the presence of bupivacaine, as shown in A (n = 7). ***p ≤ 0.001, paired Student's *t*-test. **(C)** Representative perforated whole-cell recordings from a TG neuron at a holding potential of −60 mV. Cells were exposed to bradykinin (“Bk”; 0.5 µM; cyan bar) followed by applications of capsaicin (“Cap”; 1 µM; red bar) (n = 5). **(D)** Representative traces (n = 11–18) in which capsaicin (“Cap”) was applied for 20 s in sub-EC_50_ (0.02 µM; yellow; top trace), EC_50_ (0.2 µM; orange; middle trace) and saturating (2 µM; red; bottom trace) concentrations. **(E)** Box and whiskers plots are showing the mean number of evoked action potentials (“APs (#)”; upper) and membrane potential at the end of agonist application (as indicated by an arrow in **D**; bottom) in response to the different capsaicin concentrations (n = 11–18). **p ≤ 0.01 and ***p ≤ 0.001 and ns- no statistical significance, ANOVA with multiple comparisons.

The properties of membrane depolarization affect the activity of ion channels that participate in the generation of action potentials (Bean, 2007). To examine the possibility that the different patterns of vanilloids-mediated neuronal activation result from differences in membrane depolarization, we measured voltage changes in TG neurons exposed to bradykinin or capsaicin (**Figure 4A**). To prevent the generation of action potentials and depolarization evoked by voltage-gated sodium channels, nociceptors were treated with bupivacaine throughout the analysis (Scholz et al., 1998). As summarized in **Figure 4B**, while capsaicin evoked a robust membrane depolarization (ΔVcapsaicin = 52 ± 7 mV), similarly to previous reports (Liu et al., 2001; Blair and Bean, 2003), exposure to bradykinin resulted in a moderate response (ΔV bradykinin = 17 ± 5 mV). Therefore, our analysis indicates that endovanilloids evoke close-to-threshold neuronal depolarization, which is in contrast to the robust neuronal depolarization evoked by capsaicin. Thus, our findings indicate that endo- and exovanilloids induce different levels of neuronal membrane depolarization. The observed membrane depolarizations suggest the induction of different current amplitudes by endo- and exovanilloids. To test this possibility, we measured currents in TG neurons under voltage clamp and found that while capsaicin evoked a typical robust current, endovanilloids downstream of bradykinin evoke lower current amplitude (**Figure 4C**). To test whether lowering capsaicin concentration could mimic the endovanilloids -evoked response, we measured neuronal firing in response to saturating (2 µM), EC_50_ (0.2 µM), and sub-EC_50_ (0.02 µM) capsaicin concentrations (Hazan et al., 2015; Kumar et al., 2016). Of note, we previously showed that 0.02 µM is at the lower limit of capsaicin that evokes TRPV1 activation in rat TRPV1 (rTRPV1) (**Figure S2**) (Hazan et al., 2015). As shown in **Figures 4D, E**, lowering capsaicin concentration allowed the development of more action potentials. However, bradykinin application evoked ~3-fold more action potentials as compared to capsaicin, even when the latter was applied at a sub-EC_50_ concentration (compare **Figure 1B** to **4D** and **1D** to **4E**). Importantly, for all analyzed capsaicin concentrations, depolarization block was reached. Therefore, our findings indicate that the different properties of endo- and exovanilloids, as well as their concentration, dictate the different neuronal response. Nonetheless, most exovanilloids produce depolarization block in neurons, while endovanilloids do not.

## Discussion

Endo- and exovanilloids are associated with distinct pain sensations. Here, we show that these vanilloids promote different neuronal responses. Our findings indicate that while exovanilloids produce robust but acute neuronal firing, endovanilloids–evoked TRPV1 activation supports a prolonged neuronal response (**Figure 1B**). In this work, we applied the inflammatory mediator, bradykinin, to rat TG neurons in order to induce the biosynthesis of endovanilloids intracellularly. Also, electrophysiological recordings were performed in perforated neurons in which the intracellular environment required for the production of these TRPV1 activators is intact. This experimental design better simulates the physiological conditions in which endovanilloids activate TRPV1 in comparison to either the direct application of these bioactive lipids or recordings using configurations that cause the dilution of the intracellular milieu. In our experimental settings, endovanilloids evoke lower neuronal membrane depolarization, which is sufficient to evoke firing yet does not develop into the depolarization block associated with the potent exovanilloids (**Figures 1B**, **2**, and **4A**). The weaker depolarization evoked by endovanilloids reflects their reduced activation of TRPV1 in comparison to capsaicin (**Figure 4C**) (Kumar et al., 2017). Also, we found that the reduced TRPV1 activation evoked by low capsaicin concentrations produces longer firing reminiscent of the effect of endovanilloids. However, even in sub-EC_50_ capsaicin concentrations, the neuronal firing is eventually terminated by a depolarization block, suggesting that additional molecular mechanisms may underlie TRPV1-induced neuronal firing (**Figure 4C**). Thus, we propose that the different profiles of TRPV1 activation by endo- and exovanilloids and subsequent membrane depolarization may determine the duration, and potentially the intensity, of the evoked pain response.

Our results demonstrate that individual TG neurons can produce diverse firing patterns in response to different stimuli. Indeed, the different patterns of pain sensation evoked by endo- and exo-vanilloids may reflect their contradictory functions. The prototypical, exovanilloids, capsaicin, and resiniferatoxin (RTX), are phytotoxins believed to have evolved as plants' defense mechanism as they induce a robust response by harnessing central functional protein sites (such as the TRPV1 VBS) (Cromer and McIntyre, 2008; Bohlen et al., 2010; Cao et al., 2013). As such, exposure to these toxins produces acute and intense pain. In contrast, endovanilloids are essential components in the process through which tissue injury and inflammation are detected and resolved, which requires tight regulation of their concentration and activity (Hwang et al., 2000; Hylands-White et al., 2017; Kumar et al., 2017). Thus, we propose that while exo- and endovanilloids act through the same binding site in TRPV1, they produce distinct neuronal activations that underlie their different painful effects.

It is noteworthy that not all exovanilloids produce robust TRPV1 activation and pungency. Namely, capsaicin analogs were found to cause desensitization of sensory neurons without evoking a pungent sensation (Jancsó-Gábor et al., 1970). In addition, the synthetic exovanilloids, olvanil, arvanil, and SDZ249-665, were shown to evoke little or no pungency when applied to the eye in guinea pigs (Ursu et al., 2010). While the exact mechanism of this lack of pungency remains unclear, it was shown that non-pungent exovanilloids evoke slower kinetics of TRPV1 activation and depolarization, resulting in lower probability for AP generation (Ursu et al., 2010; Viana, 2011). The reduced rate of TRPV1 activation was suggested to stem from slow penetration through the membrane, further supporting our experimental design of inducing endovanilloid synthesis intracellularly (Ursu et al., 2010). Nonetheless, we show here that slowly developing depolarization induced by TRPV1 activation allows for the recovery of Na_V_ channels, resulting in prolonged AP firing (**Figures 2** and **4**). Regardless, these findings demonstrate that the kinetics of the activation profile of TRPV1 is a significant factor affecting neuronal firing and consequent response.

We have previously shown that the submaximal activation of TRPV1 by endovanilloids stems from a low open probability of the channel compared to capsaicin–evoked activation (Kumar et al., 2017). Another TRPV1 activator which elicits reduced TRPV1 activation is the peptide spider toxin, double-knot toxin (DkTx), as it locks the channel in a sub-conducting state (Geron et al., 2018). The injection of DkTx-containing venom following this spider's bite produces prolonged pain that was attributed to the persistent activation of TRPV1 by this toxin (Liang, 2004; Bohlen et al., 2010). It is also possible that the lower TRPV1 activation by this toxin evokes weaker depolarization and avoidance of depolarization block that facilitates a continuous pain sensation. However, this spider's venom was also reported to produce an immediate intense pain similarly to capsaicin (Liang, 2004). While effects of the isolated DkTx on the neuronal and behavioral levels were not yet thoroughly tested, this toxin further demonstrates the ability of different TRPV1 activators to produce various activation profiles which may underlie distinct pain sensations.

Bradykinin affects ion channels other than TRPV1, such as ANO1 and Kv7 channels, to produce excitation in nociceptors (Liu et al., 2010). Nonetheless, as demonstrated in **Figure 3A**, the TRPV1 inhibitor, capsazepine, completely inhibits AP firing. Thus, at least in TRPV1^+^ neurons, TRPV1 activation by endovanilloids is necessary for the generation of AP firing. This does not preclude other effects induced by BK2 that may cause other changes in the firing properties of these neurons (Choi and Hwang, 2018). These effects produced by the bradykinin receptor are mainly Gq dependent, a signaling pathway that is also activated by other inflammatory mediators and is required for the endovanilloid activation of TRPV1 (Choi and Hwang, 2018). Thus, the effects of bradykinin on other ion channels are physiologically relevant and are part of TRPV1 activation by endovanilloids during inflammation.

Overall, our results suggest that the regulated activity of TRPV1 by endovanilloids allows a prolonged, low-frequency neuronal response. When toxins (such as capsaicin) bypass this regulation, neuronal membrane depolarization block is achieved, and the response is rapidly terminated. Thus, although capsaicin is a powerful tool to study this pivotal pain receptor, its activity may not reproduce the endogenous receptor response. The hydrophobicity of endovanilloids and the transient nature of their synthesis make them challenging to investigate (Starowicz et al., 2007). Nevertheless, a full understanding of TRPV1's physiological role necessitates studying its response to multiple endogenous stimuli. Elucidating the mechanisms through which inflammatory pain is generated may facilitate the identification of new targets as well as the development of new tools for pain management.

## Data Availability Statement

The datasets generated for this study are available on request to the corresponding author.

## Ethics Statement

The animal study was reviewed and approved by Ethics Committee of the Hebrew University.

## Author Contributions

AP designed research. RK, MG, and AH performed research. RK, MG, AH, and AP analyzed data. MG and AP wrote the paper.

## Funding

This work was supported by the Israel Science Foundation (Grant 1444/16 to AP), the Brettler Center and David R. Bloom Center, School of Pharmacy (The Hebrew University of Jerusalem; to AP), a Jerusalem Brain Committee Postdoctoral Fellowship (to RK) and the Paula Goldberg fellowship (to MG).

## Conflict of Interest

The authors declare that the research was conducted in the absence of any commercial or financial relationships that could be construed as a potential conflict of interest.
