# Supplementary material for: Endogenous and Exogenous Vanilloids Evoke Disparate TRPV1 Activation to Produce Distinct Neuronal Responses
Source: Front Pharmacol. 2020 Jun 12;11:903. doi: 10.3389/fphar.2020.00903 (PMC7303340; doi:10.3389/fphar.2020.00903)
Supplement: Supplementary file 1 [file DataSheet_1.pdf]

Supplementary Material

Supplementary Figures

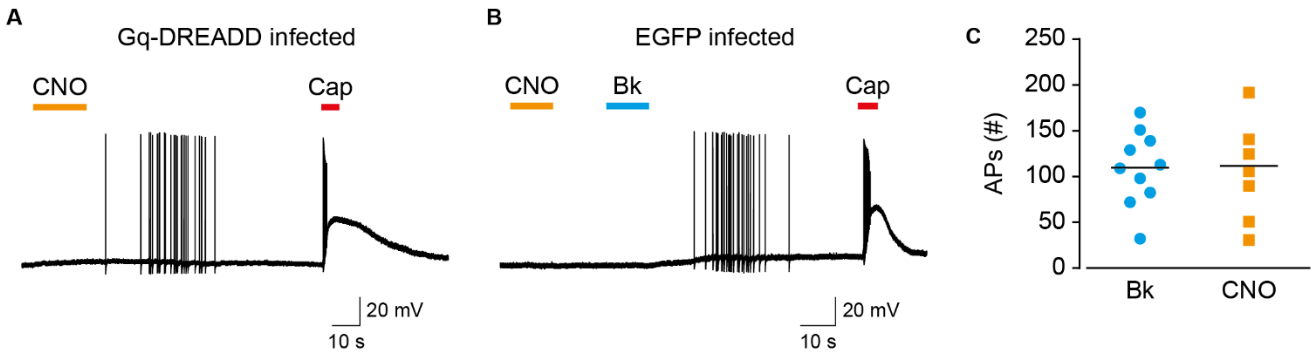

Supplementary Figure 1. Gq-DREADD mimics the bradykinin response in infected sensory neurons. Current-clamp recordings (I=0) from small-diameter dissociated rat trigeminal ganglion (TG) neurons (P0) using the perforated whole-cell configuration of the patch-clamp technique. **(A)** ***Top***: Neurons infected with AAV-hSyn-HA-hM3D(Gq)-IRES-mCitrine ('Gq-DREADD infected') were exposed to clozapine-n-oxide ('CNO'; 5 μM) for 15 s (orange bar) followed by capsaicin ('Cap'; 0.2 μM) application for 5 s (n=7). ***Bottom***: Neurons infected with AAV-hSyn-EGFP ('EGFP infected') were exposed to clozapine-n-oxide ('CNO'; 5 μM) for 10 s (orange bar) followed by bradykinin ('Bk'; 0.5 μM) application for 10 s (cyan bar) and then capsaicin ('Cap'; 0.2 μM) application for 5 s (n=10). **(C)** Mean/scatter-dot plot representing the number of action potentials (APs) of EGFP infected neurons in response to bradykinin ('Bk'; 0.5 μM; cyan circles) and Gq-DREADD-infected neurons in response to clozapine-n-oxide ('CNO'; 5 μM; orange squares) (n=7-10).

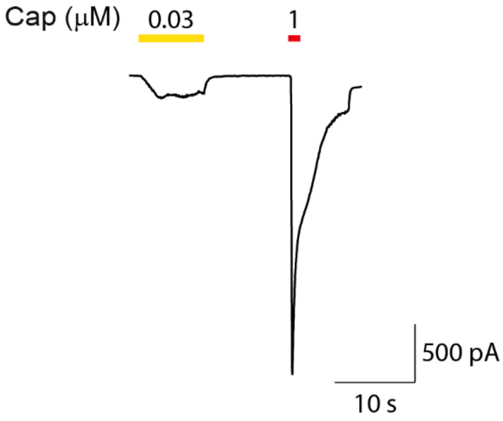

Supplementary Figure 2. 0.03 μM is a sub-EC$_{50}$ concentration of capsaicin. Representative perforated whole-cell recording from a TG neuron at a holding potential of −60 mV. Cells were exposed to 0.03 μM capsaicin (yellow bar) followed by applications of 1 μM capsaicin (red bar) (n=3).
